# Molluscicidal activity of various solvent extracts from *Solanum nigrum* var. *villosum* L. aerial parts against *Galba truncatula*

**DOI:** 10.1051/parasite/2011181063

**Published:** 2011-02-15

**Authors:** H. Hammami, R. Mezghani-Jarraya, M. Damak, A. Ayadi

**Affiliations:** 1 Fungal and Parasitic Molecular Biology Laboratory, Faculty of Medicine, University of Sfax 3029 Sfax Tunisia; 2 Laboratory of Chemistry of Natural Products, Faculty of Science, University of Sfax Tunisia

**Keywords:** molluscicidal activity, *Galba truncatula*, *Fasciola hepatica*, *Solanum nigrum* var. *villosum* L., alkaloids, saponosides, molluscicide, *Galba truncatula*, *Fasciola hepatica*, *Solanum nigrum* var. *villosum* L., alcaloïdes, saponosides

## Abstract

Molluscicidal activity of *Solanum nigrum* var. *villosum* (morelle velue) extracts and their fractions were tested against the mollusca gastropoda *Galba truncatula* intermediate host of Fasciola hepatica. The results indicated that the hydro-methanol (MeOH-H_2_O) immature fruit extract possess the highest molluscicidal activity (LC_50_ = 3.96 mg/L) against *Galba truncatula* compared with other tested compounds. After acido-basic treatment, the methanolic extract fraction isolated from the immature fruits and the richest in alkaloids was the most toxic (LC_50_ = 1.65 mg/L). The fractions richest in saponosides obtained from the hydromethanolic and methanolic extracts of immature fruits showed interesting molluscicidal activities (LC_50_ = 6.15 mg/L and LC_50_ = 7.91 mg/L, respectively). The observed molluscicide activity could be attributed to the presence of alkaloids or saponosides. So, the immature fruits of *Solanum nigrum* var. *villosum* could be substrates of choice for molluscicide activity. In addition, total alkaloids and saponosides present in this plant deserve further investigations in order to identify the active principles and demonstrate their activities on mollusks in their natural habitat. According to the World Health Organization’s guidelines on screening for plant molluscicides, use of these fractions may add to the arsenal of methods to control snail transmitting fasciolosis in tropical and Third World countries where fasciolosis is a common disease.

## Abstract (fr)

Les activités molluscicides des extraits au dichlorométhane, au méthanol et au méthanol-eau des différents organes (feuilles, fruits immatures et fruits mâtures) de *Solanum nigrum* var. *villosum* (morelle velue) ont été testées contre le mollusque gastéropode *Galba truncatula* hôte intermédiaire de Fasciola hepatica. Les résultats ont montré que l’extrait hydrométhanolique (MeOH-H_2_O) des fruits immatures était le plus actif contre le mollusque *Galba truncatula* (CL50 = 3,96 mg/L) comparativement aux autres composés testés. Après traitement acido-basique, la fraction de l’extrait méthanolique, isolée à partir des fruits immatures et la plus riche en alcaloïdes, était la plus toxique (CL50 = 1,65 mg/L). De plus, les fractions les plus riches en saponosides des extraits hydrométhanoliques et méthanoliques des fruits immatures ont montré des activités molluscicides intéressantes (CL50 = 6,15 mg/L et CL50 = 7,91 mg/L, respectivement). L’activité molluscicide observée pourrait donc être attribuée à la présence d’alcaloïdes ou de saponosides. Ainsi, les fruits immatures de *Solanum nigrum* var. *villosum* pourraient être des substrats de choix pour l’activité molluscicide. De plus, les alcaloïdes totaux et les saponosides présents méritent des investigations supplémentaires afin d’identifier les principes actifs et prouver leurs activités sur les mollusques dans leur habitat naturel. Selon l’OMS, l’utilisation de ces fractions pourrait s’ajouter à l’arsenal des méthodes de lutte contre les mollusques transmettant la fasciolose dans les pays tropicaux et le Tiers-Monde où la fasciolose est endémique.

Fasciolosis caused by *Fasciola hepatica* (*F. hepatica*), a parasite trematode is of considerable medical and veterinarian importance. This old disease has a great potential of expansion in view of the large colonization capacities of its fasciolid causal agents as well as the freshwater lymnaeid snail vector species (Mas-Coma *et al.*, 2001). The control of Fasciolosis depends on the understanding of the epidemiology of the disease, especially the biology, ecology and distribution of the snails that act as intermediate hosts. It is generally considered that snail control by mollusciciding is of considerable medical and veterinarian importance and can be a rapid and effective means of reducing Fasciolosis transmission (Madsen, 1985). The search for local plants with molluscicidal activity was considered more sustainable than the use of synthetic molluscicides. Plants from Solanaceae family with several species of *Solanum* have been found to have a significant and potentially useful molluscicidal activity (Medina & Ritchi, 1980; Kloos *et al.*, 1987; Beccouche et al., 2000; Wei *et al.*, 2002; Silva *et al.*, 2006). *Galba truncatula* (*G. truncatula*) has been identified as the main intermediate host of fasciolosis in southern Tunisia (Ayadi *et al.*, 1997, Hammami & Ayadi, 1999; Hammami *et al.*, 2007). Previous studies in our laboratories have shown the high molluscicidal activity of alkaloids especially the N-methylisosalsoline isolated from *Hammada scoparia*, Chenopodiaceae (Jarraya *et al.*, 2009). It was also reported in earlier studies that crude water extracts of *Solanum nigrum* var. *villosum* has molluscicidal and antiparasitic activity against the mollusca gastropoda *G. truncatula* (Hammami & Ayadi, 2008).

In our on going researches for the isolation and the purification of more plant molluscicidal active products, we aimed our study to explore molluscicidal activities of various solvent extracts and fractions from *Solanum nigrum* var. *villosum* aerial parts against *G. truncatula*.

## Material and Methods

### Plant Material

*Solanum nigrum* var. *villosum* L. 1753, a common annual herbaceous weed (Solanaceae family) locally known as “Enab-El-Dheeb”, was described in many fields of the south- western of Tunisia by Pottier Alapetite (1981). Samples of this plant were collected in June 2006 from Tozeur’s traditional oases, Tunisia and botanically identified by Prof. Ridha Krichen, Botanist at “École d’Horticulture de Chott Mariem, Sousse, Tunisie”. Voucher specimens (LFPMB10) have been deposited at the “Fungal and Parasitic Molecular Biology Laboratory, Faculty of Medicine, Sfax, Tunisia”. Leaves, ripe red and unripe green fruits collected in Tozeur, Tunisia, were carefully detached from the fresh plant and air-dried.

### Extractions

Air-dried leaves, ripe red and unripe fruits of *Solanum nigrum* var. *villosum* were extracted (during 24 hours each time) successively in a Soxhlet apparatus by solvents of increasing polarity: dichloromethane, methanol and then lixiviated with methanol-water (8:2, v-v) at room temperature.

All extracts were filtered through paper Whatman No.1 and then concentrated under reduced pressure to afford the dichloromethane extract (DE) and the methanolic extract (ME). For the methanol-water (8:2, v-v) extract, methanol was removed under reduced pressure and the remaining aqueous phase was lyophilized to afford MeOH-H_2_O extract. Each extract was weighed (Table I) and kept in dark at 4 °C until use.

**Table I.** Mass and yields of different plant part extracts of *Solanum nigrum* var. *villosum* after 24 hours extraction with increasing polarity solvents.

	Leaves (114 g)		Mature fruits (400 g)		Immature fruits (200 g)	
Initial mass of plant parts (g)	Mass (g)	Yield (%)	Mass (g)	Yield (%)	Mass (g)	Yield (%)
Dichloromethane extract (CH_2_Cl_2_)	4.21	3.69	0.40	0.1	0.18	0.09
Methanol extract (MeOH)	18.06	15.84	21.72	5.43	8.31	4.15
MeOH-H_2_O extract (8:2, v-v)	0.05	0.04	2.45	0.61	0.24	0.12

### Phytochemical Screening

The preliminary phytochemical screening was performed according to the Harborne (1964, 1973) methods. Plant extracts (dichloromethane, methanol and methanol-water) were subjected to chemical tests for the presence of sterols, triterpenoids, carotenoids, tropolone, quinones, alkaloids, flavonoids and saponosides.

A sample of each extract or fraction obtained is dissolved in a minimum amount of solvent adequate for a solution E. This will be added to various reagents according to the protocols experimental T_1_, T_2_, T_3_, T_4_, T_5_ and T_6_.

T_1_: reaction of Liebermann: 1 ml E + 0.2 ml of acetic anhydride + 4 drops of H_2_SO_4_ (cc). Violet changing to blue-green indicates the presence of sterols and (or) triterpenoids.

T_2_: reaction of Carr and Price: 1 ml E + 4 drops of SbCl_3_ 20% in CHCl_3_. Coloring fleeting blue to blue violet could be a sign of the presence of carotenoids and (or) triterpenoids.

T_3_: reaction of Wiustater: 1 ml of E + 0.2 ml of MeOH + 1 drop of FeCl_3_ (0.005 M) + 0.6 ml water + 0.4 ml of CHCl_3_. Red coloration of the chloroform layer indicates the presence of a tropolone nucleus.

T_4_: reaction of Borntraeger: 2 ml E + 2 ml of NaOH (0.1 M). If the aqueous phase is colored of red to violet, we can deduce the presence of free quinons.

T_5_: test of flavonoids: 1 ml of E + 2 ml EtOH-H_2_O (1-1) + some drops of HCl + Mg chips. Orange to red-violet coloration and blue indicates the presence of flavonoids.

T_6_: Mayer reaction: 1 ml of E + 0.5 ml HCl (0.1 N) + 5 drops of Mayer reagent. Formation of a white precipitate indicates the presence of alkaloids.

The result with a single reagent being not absolute because there are false positive, we used a second reagent for alkaloids: the Dragendorff reagent revealing red-orange spots of alkaloids on thin layer chromatography plates.

. Mayer reagent: (solution A: 13.5 g HgCl_2_ + 20 ml water and solution B: 49.8 g KI + 20 ml water). The two solutions are mixed and diluted with water to one liter.

. Dragendorff reagent: formula Munier and Macheboeuf for alkaloids: (solution I: dissolve 0.85 g of under bismuth nitrate in a mixture of 10 ml glacial acetic acid + 40 ml water; solution II: 8 g KI + 20 ml water). Immediately before use, mix 15 ml of solution I and 15 ml of solution II, and add 20 ml of glacial acetic acid and make up to 100 ml with water. On TLC, the total steroidal saponins appear red-purple after spraying a solution of chloroform saturated antimony trichloride, and few minutes after heating the plate to 100-110 °C. All steroidal nuclei are located without distinguishing saponosides and glycoalkaloids (GA). The glycoalkaloids (GA) were revealed as orange spots on TLC by Dragendorff reagent.

The concentration of the chemical groups investigated was scored: - (no reaction), + (weakly positive reaction), ++ (important positive reaction), +++ (very important positive reaction).

### Fractionation of Active Extracts

An acido-basic treatment of each active extract (except hydromethanolic extract of leaves) was performed as follows: m(g) of each extract were solubilized in hydrochloric solution (0.05 M) then extracted thrice by ethyl acetate (EtOAc). Following evaporation of the ethyl acetate, we obtained the extract A (m_A_). The aqueous layer was alkalinized by addition of ammoniac then extracted by ethyl acetate until negative Mayer test. After evaporation of the ethyl acetate, we obtained the extract B (m_B_) containing total alkaloids revealed as orange spots on TLC by Dragendorff reagent. Then, the remaining aqueous layer was extracted thrice by butanol. After evaporation of the butanol, we obtained the extract C (m_C_) containing polar products such as saponins, which were revealed as red-purple spots on TLC by SbCl_3_/CHCl_3_ (Aubert *et al.*, 1989). Finally, the remaining aqueous layer was lyophilized to produce the extract D (m_D_).

### Snails

Uninfected adult *G. truncatula* (3-5 mm in length) were collected locally from a deep draining canal isolated from definitive host, located in the Tozeur’s traditional oases in February 2007 and transferred in laboratory aquaria and acclimatized for a minimum period of four days, in holding tanks containing aerated, dechlorinated tap water and washed sand, before being exposed to the aqueous extracts. The toxicity of these preparations was also tested on *G. truncatula* control organisms.

### Molluscicidal Tests

Evaluation of the molluscicidal activity of *Solanum nigrum* var. *villosum* extracts and CuCl_2_ (used as positive control) against snails was investigated as recommended by the World Health Organization (1965). The CuCl_2_ solution was prepared by adding dechlorinated water to 1 g of CuCl_2_ up to 1 liter. The extract or fraction of each plant part and of CuCl_2_ was used to produce a series of concentrations so that the molluscicidal potency of each sample could be evaluated. The snails were exposed, in groups of ten (five replicates) for 24 to 48 hours, to 500 ml of each concentration of one of the tested materials: leaf, ripe red and unripe green fruit extracts, or CuCl_2_ (positive control product) shown in Table III.

*Galba truncatula* negative control organisms were similarly immersed in dechlorinated water. After exposure, snails were rinsed thoroughly in dechlorinated water and left for 48 hours in dechlorinated water before mortality was evaluated.

Mortality was recorded after 24 to 48 hours and dead animals were removed immediately to avoid contamination of animals. Snail mortality was established by the contraction of the body within the shell; no response to a needle probe was taken as evidence of death.

### Statistics

The concentrations that would kill 50% (LC_50_), 90% (LC_90_) of the exposed snails and the confidence interval (95% CI), were determined by the R language analysis which is an integrated suite of software facilities for data manipulation, calculation and graphical display (Ihaka & Gentleman, 1996).

## Results

The mass and yields of different plant part extracts of *Solanum nigrum* var. *villosum* after 24 hours extraction with increasing polarity solvents were given in Table I.

The phytochemical tests performed for alkaloids and saponosides were positive for the methanolic extracts and important for the MeOH-H_2_O (8:2, v-v) extracts. We detected sterols and terpenoids in dichloromethane extracts. The flavonoids were present in the hydromethanolic extracts (Table II).

**Table II.** Chemical groups present in the extracts from aerial parts of *Solanum nigrum* var. *villosum*.

Phytochemical test		T_1_ (sterols; triterpenoids)	T_2_ (carotenoids; triterpenoids)	T_3_ (tropolone)	T_4_ (quinone)	T_5_ (flavonoids)	T_6_ (alkaloids)	(saponosides)
	Leaves	+	+	–	–	–	–	–
Dichloromethane	Mature fruits	+	+	–	–	–	–	–
	Immature fruits	+	+	–	–	–	–	–

	Leaves	–	–	–	–	–	+	+
Methanol	Mature fruits	–	–	–	–	+	–	–
	Immature fruits	–	–	–	–	–	+	+

	Leaves	–	–	–	–	+	+	+
Methanol-H_2_O (8:2, v-v)	Mature fruits	–	–	–	–	+	+	++
	Immature fruits	–	–	–	–	+	++	++

- : no reaction; + : weakly positive reaction; ++ : important positive reaction.

The molluscicidal activities of *Solanum nigrum* var. *villosum* extracts and the cupric chloride against the mollusca *G. truncatula* were given in Table III. Toxic activity of this plant was determined for dichloromethane, methanolic and hydro-methanolic extracts.

**Table III.** Molluscicidal activities of *Solanum nigrum* var. *villosum* extracts, after 48 hours exposure time, against *Galba truncatula*.

Extract	Leaves		Mature fruits		Immature fruits	
	LC_50_ (mg/L) (95% CI)	LC_90_ (mg/L) (95% CI)	LC_50_ (mg/L) (95% CI)	LC_90_ (mg/L) (95% CI)	LC_50_ (mg/L) (95% CI)	LC_90_ (mg/L) (95% CI)
Dichloromethane (CH_2_Cl_2_)	31.37 (27.50; 35.24)	46.53 (39.12; 53.94)	50.84 (45.53; 58.15)	64.8 (48.04; 81.21)	34.41 (30.64; 38.18)	50.47 (43.97; 56.97)

Methanol (MeOH)	17.51 (14.86; 20.16)	26.05 (21.97; 30.13)	0	0	15.19 (14.38; 96.19)	18.85 (17.44; 20.26)

MeOH-H_2_O extract (8:2, v-v)	15.92 (13.43; 18.41)	23.45 (19.63; 27.27)	84.83 (43.28; 26.38)	152.36 (50.32; 254.39)	**3.96** (3.11; 4.81)	**7.49** (6.18; 8.8)

Dechlorinated water	0	0	0	0	0	0

LC_50_: 50% lethal concentration; LC_90_: 90% lethal concentration; CI: confidence interval.

CuCl_2_ showed molluscicidal activity against the mollusca gastropoda *Galba truncatula* after 48 hours with LC_50_ = 26.12 (19.35; 31.69) mg/L and LC_90_ = 62.71 (49.83; 96.50) mg/L; and after 144 hours with LC_50_ = 6.20 (5.42; 6.94) mg/L and LC_90_ = 11.34 mg/L.

The hydro-methanolic extract of the immature fruits, after 48 hours, showed the highest activity with values for LC_50_ = 3.96 mg/L and LC_90_ = 7.49 mg/L. By comparison, we obtained values of LC_50_ = 15.19 mg/L and LC_90_ = 18.85 mg/L for the methanolic extract and LC_50_ = 34.41 mg/L and LC_90_ = 50.47 mg/L for the dichloromethane extract.

For leaves, the hydro-methanolic extract (LC_50_ = 15.92 mg/L) and the methanolic extract (LC_50_ = 17.51 mg/L) showed interesting toxicity compared with the dichloromethane extract (LC_50_ = 31.37 mg/L).

Mature fruit extracts show the lowest toxicities with LC_50_ = 50.84 mg/L for the dichloromethane extract and LC_50_ = 84.83 mg/L for the hydro-methanolic extract. The methanolic extracts of mature fruits were inactive.

Cupric chloride, used as a reference substance, killed 90% of mollusks at 11.34 mg/L after six days of treatment. Control assays with dechlorinated water showed no effect on the snails (Table III). Haemolysis and hyper secretion of mucus were the common toxic responses of snails to the active tested materials.

The molluscicidal activity of methanolic and hydromethanolic extracts of *Solanum nigrum* after acidobasic treatment were reported in Table IV.

**Table IV.** Yields, phytochemical tests and molluscicidal activities of *Solanum nigrum* var. *villosum* fractions, after 24 and 48 hours exposure time, against *Galba truncatula*.

				Activity after 24 H exposure time		Activity after 48 H exposure time		Phytochemical tests	
Initial mass (mi) of extract (g)	Solvant / pH	Mass (mg)	Yields (%)	LC_50_ (mg/L) (95% CI)	LC_90_ (mg/L) (95% CI)	LC_50_ (mg/L) (95% CI)	LC_90_ (mg/L) (95% CI)	Dragendorff	SbCl_3_
	EtOAc / acid medium	m_A_ = 96	4.7	inactive	inactive	inactive	inactive	–	–
MeOH-H_2_O / Mature fruits mi = 2.02	EtOAc / alkali medium	m_B_ = 20	1	inactive	inactive	inactive	inactive	+	–
	Butanol / alkali medium	m_C_ = 288.2	14.25	15.54 (6.39; 37.75)	28.30 (20.42; 39.23)	14.29 (5.98; 34.10)	27.37 (14.79; 50.66)	–	++
	Aqueous lyophilized layer	m_D_ = 1,440	71.28	inactive	inactive	inactive	inactive	–	–

	EtOAc / acid medium	m_A_ = 182.1	26.38	inactive	inactive	inactive	inactive	–	–
MeOH / Leaves mi = 0.69	EtOAc / alkali medium	m_B_ = 3.6	0.52	inactive	inactive	inactive	inactive	+	–
	Butanol / alkali medium	m_C_ = 73	10.57	12.59 (2.61; 60.59)	22.76 (11.88; 43.59)	13.64 (2.28; 81.47)	24.36 (17.46; 34.00)	–	++
	Aqueous lyophilized layer	m_D_ = 340	49.27	inactive	inactive	inactive	inactive	–	–

	EtOAc / acid medium	m_A_ = 26.5	0.18	inactive	inactive	inactive	inactive	–	–
MeOH-H_2_O / Immatures fruits mi = 0.15	EtOAc / alkali medium	m_B_ = 6.7	4.47	inactive	inactive	inactive	inactive	+	–
	Butanol / alkali medium	m_C_ = 23.6	15.73	10.96 (1.31; 91.61)	36.38 (19.32; 68.51)	6.15 (0.10; 347.89)	18.16 (2.83; 116.43)	–	+++
	Aqueous lyophilized layer	m_D_ = 90	60	inactive	inactive	inactive	inactive	–	–

	EtOAc / acid medium	m_A_ = 81.8	12.58	inactive	inactive	inactive	inactive	–	–
	EtOAc / alkali medium	m_B_ = 11.1	1.7	**2.56** **(0.78; 8.46)**	7.56 (3.44; 16.61)	**1.65** **(0.61; 4.45)**	4.77 (0.83; 27.45)	+++	–
MeOH / Immatures fruits mi =0.65	Butanol / alkali medium	m_C_ = 181.6	27.94	14.69 (4.09; 52.71))	48.07 (21.10; 109.51)	7.91 (0.27; 234.79)	26.20 (7.99; 85.86)	–	+++
	Aqueous lyophilized layer	m_D_ = 340.2	52.34	24.59 (1.13; 536.74)	89.40 (5.44; 146.93)	20.46 (3.11; 134.43)	55.26 (17.89; 170.61)	–	+

Dechlorinated water				0	0	0	0		

LC_50_: 50% lethal concentration; LC_90_: 90% lethal concentration; CI: confidence interval.

CuCl2 showed molluscicidal activity against the mollusca gastropoda *Galba truncatula* after 48 hours with LC_50_ = 26.12 (19.35; 31.69) mg/L and LC_90_ = 62.71 (49.83; 96.50) mg/L; and after 144 hours with LC_50_ = 6.20 (5.42; 6.94) mg/L and LC_90_ = 11.34 mg/L.

The highest toxicity, after 48 hours, was shown with immature fruit fractions: the fractions B (LC_50_ = 1.65 mg/L) and C (LC_50_ = 7.91 mg/L) of the methanolic extract and the fraction C (LC_50_ = 6.15 mg/L) of the hydromethanolic extract.

Weaker molluscicidal activities were obtained for the fraction C of leaf methanol extract (LC_50_ = 13.6 mg/L) and mature fruit hydromethanolic extract (LC_50_ = 14.29 mg/L).

The molluscicidal activity disappeared for the fraction A of each fractionated extract.

## Discussion

One of the major preventive steps against fasciolosis is the control of the vector snail populations. In recent years, several plant extracts have been studied against snail transmitted parasitic diseases and several species of *Solanum* have been found to have significant and potentially useful molluscicidal activity. Such activity varies greatly from species to species and even between different parts of the same plant (Silva *et al.*, 2005).

During our study, only immature fruit and leaf extracts showed significant molluscicidal activities with LC50 values that fell well below the upper threshold of 40 mg/L set for a potential molluscicide by the World Health Organization (WHO, 1993). So that, the immature fruit extracts have the most toxicity against the snail *G. truncatula* correlating with previous studies (Hammami & Ayadi, 2008). By comparison with immature fruit crude water extract of the same plant (*Solanum nigrum* var. *villosum* collected from Tozeur’s oases) obtained values (LC_50_ = 41.2 mg/L and LC_90_ = 66.6 mg/L) in our previous studies, the immature fruit hydro-methanolic extract was more efficacious and demonstrated the importance of the plant components separation.

This result was even supported by Ndamukong *et al.* (2006) when studying molluscicidal activity of *Solanum scabrum* against the snails *Bulinus camerunensis* and *B. truncatus* showing that methanolic extracts were more toxic than water extracts.

Methanolic extract was found even possessing a strong molluscicidal activity and exhibited potent effect on mollusks (Lahlou *et al.*, 2002).

In the present study, methanolic and hydro-methanolic extracts of the aerial green parts (leaves and immature fruits) were the most toxic from the whole extracts tested. These findings are in agreement with other data in the literature. For instance, aerial parts methanolic extracts of *Solanum jabrense* were described to have molluscicidal activity with LC_50_ = 56.0 mg/L and LC_90_ = 80.3 mg/L (Silva *et al.*, 2006).

It was even reported that the methanolic extract of the berries of *Solanum aculeastrum* also showed significant activity against host snails of schistosomiasis, the LC_100_ = 50 mg/L (Wanyonyi *et al.*, 2003).

According to other studies, the cercaricidal activity was related to the flavonoids, terpenoids, and saponosides (Chifundera *et al.*, 1993; Perret & Whitefield, 1995; Lahlou *et al.*, 2002). It was also reported that the toxicity in *Solanum genera* was accorded to the presence of flavonoids (Silva *et al.*, 2002) and steroidal alkaloids in the fruits (Bhattacharyya, 1984; Valverde *et al.*, 1993). Alkaloids of stems of *Solanum stipulaceum* were toxic against *Biomphalaria glabrata* LC_50_ = 45.2 mg/L and LC_90_ = 56.0 mg/L (Silva *et al.*, 2006).

In our study, the fractionation has concerned only active extracts showing significant LC_50_ below the upper threshold of 40 mg/L (Table III) fixed by WHO (1993) and that were positive for alkaloid and saponosides phytochemical tests (Table II). Leaf hydromethanolic extract having a fewer yield (0.04%) was not fractionated (Table I).

During our study, after acido-basic treatment, we noticed that fractions showing molluscicidal activity contained generally alkaloid and/or saponosides (Table IV).

Among the eight different total alkaloid and saponosides fractions tested, that from immature fruit methanolic extract was the most potent in *G. truncatula* (LC_50_ = 1.65 mg/L) but it was presented in a weaker yield of 1.7% . Even in other studies, it was reported that *S. nigrum* immature fruits are not a promising source of the alkaloids because of their small size and declining alkaloid content (Eltayeb *et al.*, 1997). Other workers have reported steroidal alkaloids in *Solanum incanum* fruits at levels ranging from traces (Lin *et al.*, 1990) to 0.031% (Fukuhara & Kubo, 1991) on a fresh weight basis in mature fruits.

The fractions B less rich in alkaloids from mature fruit hydromethanolic (1%) and leaf methanolic (0.52%) extracts were not active against *G. truncatula* and that may be attributed to the fact that total alkaloids were not enough to produce molluscicidal activity.

The fraction C rich in saponosides was active in all our extracts tested and it presented important yields (ranging from 10.57% to 52.34%) and interesting molluscicidal activity. The fraction C from hydromethanolic immature fruit extract was found to be the next toxic against *G. truncatula* (LC_50_ = 6.15 mg/L) and after that was classified the fraction C from immature fruit methanolic extract (LC_50_ = 7.91 mg/L). We deduce that molluscicidal activity was related to polar fractions rich in alkaloids and /or saponosides and that the fraction richest in alkaloids from the immature fruits was more toxic than those rich in saponosides.

Saponosides fractions from leaves methanolic extract and mature fruits hydromethanolic extracts have shown weaker molluscicidal activity (LC_50_ = 13.64 mg/L and LC_50_ = 14.29 mg/L, respectively).

So, we concluded that immature fruits were best candidates for molluscicidal activity. This is in agreement with earlier studies carried out in our laboratories confirming the highest toxicity of crude water extracts of immature fruits of the same plant *Solanum nigrum* var. *villosum* (Hammami & Ayadi, 2008).

Immature fruits more rich in alkaloids were more toxic then mature fruits, this may be due to the high concentration of steroidal alkaloids. So, it was demonstrated that small unripe fruits of *Solanum nigrum* had a high concentration of solasodine that decreased with fruit maturation (Eltayeb *et al.*, 1997).

Phytochemical tests indicated the steroidal nature of the total alkaloids of *Solanum nigrum*. Several steroidal alkaloids including solasodine, solasonine and solamargine were isolated and identified in this plant (Schreiber, 1968; Ripperger & Schreiber, 1981; Drewes & Staden, 1995; Eltayeb *et al.*, 1997). Steroidal saponins were previously isolated and identified as nigrumin I and II (Tsuyoshi *et al.*, 2000). Six new steroidal saponins were also isolated by Zhou *et al.* (2006).

Hence, the observed molluscicidal activity may be due to the presence of steroidal alkaloids. It was reported that steroidal alkaloids from *Solanum aculeastrum* possess strong molluscicidal properties (Wanyonyi *et al.*, 2002). In other studies, saponins and steroidic alkaloids were isolated from *Solanum melongena* L. fruits (Aubert *et al.*, 1989).

## Conclusion

In the present study, molluscicidal activity of *Solanum nigrum* var. *villosum* varies between different parts of this plant and may be attributed to the nature of components. The analyses by thin layer chromatography achieved on the different extracts of this plant showed that the methanolic and hydromethanolic ones were the richest in alkaloids and/or saponosides and the most active against *G. truncatula*. So, molluscicidal activity may be correlated with the content of total alkaloid and/or saponosides found in the methanolic and the hydro-methanolic immature fruit extracts. This finding is in agreement with other data in the literature confirming that alkaloids from methanolic extracts of *Solanum aculeastrum* showed significant activity against host snails of schistosomiasis (LC_90_ = 50 ppm) (Wanyonyi *et al.*, 2003). However, molluscicidal activity of *Solanum nigrum* var. *villosum* collected in Tunisia was more active and especially the hydro-methanolic extract (LC_50_ = 3.96 mg/L) and the methanolic extract fraction richest in alkaloids (LC_50_ = 1.65 mg/L) isolated from the immature fruits.

Even the fractions richest in saponosides from the immature fruit hydromethanolic and methanolic extracts have shown interesting molluscicidal activity (LC_50_ = 6.15 mg/L and LC_50_ = 7.91 mg/L, respectively). In our knowledge, this is the first study proving that immature fruit methanolic or hydromethanolic fractions rich in alkaloids and/or saponosides of *Solanum nigrum* var. *villosum* have important molluscicidal activity against the snail gastropoda *G. truncatula*.

Green berries of *Solanum nigrum* var. *villosum* offers promised as a potential molluscicidal control agent and the total alkaloids and saponosides from these plant merit further investigations to identify the active principal responsible of molluscicidal properties and to further assess their activity in *G. truncatula* and its associated fauna in their natural habitats.

According to the World Health Organization’s guidelines on screening for plant molluscicides (WHO, 1983), use of these fractions may add to the arsenal of methods to control snail transmitting fasciolosis in tropical and third world countries where fasciolosis is a common disease.
